# Cheminformatics as a core driver of molecular discovery and design

**DOI:** 10.1186/s13321-026-01275-6

**Published:** 2026-10-16

**Authors:** Barbara Zdrazil, Karina Martinez-Mayorga

**Affiliations:** 1 European Molecular Biology Laboratory, European Bioinformatics Institute (EMBL-EBI), Wellcome Genome Campus, Hinxton, Cambridgeshire CB10 1SD UK; 2 Institute of Chemistry, Campus Merida, National Autonomous University of Mexico, Merida–Tetis Highway, Km. 4.5, Ucu, 97357 Yucatan Mexico

## Introduction

As the *Journal of Cheminformatics* enters a new editorial phase in 2026, we take the opportunity to revisit its vision, reconsider its scope and focus, and reflect on the evolving role of cheminformatics as a discipline over the past few years. That reflection, in turn, shapes our perspective on how the Journal can move the field of cheminformatics and Open Science forward in the coming years. We also take this opportunity to thank Dr. Rajarshi Guha, who stepped down as Co-Editor-in-Chief at the end of 2025, for his many years of dedication to the Journal and to the community it serves.

## From a bridging discipline to a core driver

In our 2022 editorial we described cheminformatics as a *bridging discipline*—one that joins chemistry and information technology to store, index, and search chemical information, and in doing so helps to solve complex problems in drug discovery and beyond [1]. Four years on, that description, while still accurate, no longer feels sufficient. Cheminformatics has evolved from a bridge between established fields to a core driver of how molecules are discovered, designed, and understood. Methods first developed within our community now underpin work in fields like, e.g., drug discovery, chemical biology, materials science, metabolomics, and environmental chemistry, and increasingly shape the questions those fields are able to ask. The need for an updated perspective reflects how the field continues to grow and mature.

This shift carries both opportunity and responsibility. As cheminformatics methods are adopted ever more widely, the standards we set as a community are becoming ever more consequential. In the age of AI, such standards forth and foremost include scientific rigour, reproducibility, and clear criteria for what constitutes a genuine contribution.

*J. Cheminform.* serves not merely as a repository of methods, but as a venue that helps define the vocabulary and the expectations of the discipline.

## A decade of evolution

**Fig. 1:**
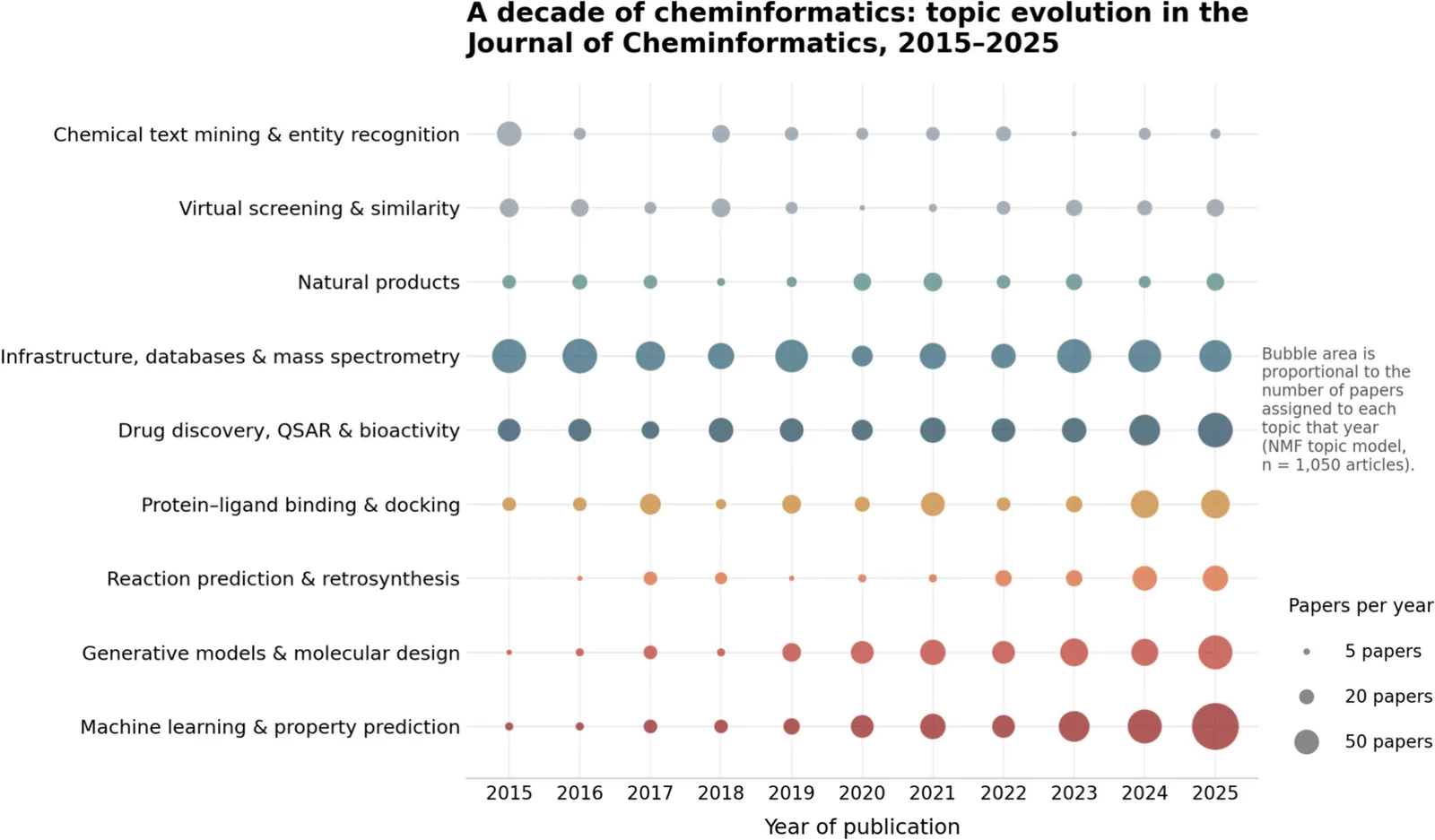
Topic evolution in the Journal of Cheminformatics, 2015–2025. Each bubble represents the number of articles assigned to a given topic cluster in a given year; bubble area is proportional to the article count. The corpus comprises 1,050 articles retrieved from PubMed using the query “Journal of Cheminformatics” [Journal] AND 2015:2025[dp]. Clusters were derived by non-negative matrix factorisation (NMF, k = 9) over a TF-IDF representation of titles (weighted 2 ×) and abstracts; each article is assigned to its dominant topic. Cluster labels were assigned post-hoc by inspection of the fourteen most characteristic terms per cluster. Code and a full method description are available at https://github.com/BZdrazil/jcheminf-editorial-2026/. The figure illustrates the rapid rise of machine learning, property prediction, and generative molecular design, alongside the steady persistence of database, infrastructure, and structure–activity research

Evidence for this view can be found in the topics published in *J. Cheminform.* over the past decade. Applying a topic model to the titles and abstracts of 1,050 articles published between 2015 and 2025 (Fig. 1), reveals a clear trajectory. Through the mid-2010s, the Journal was dominated by foundational work: chemical infrastructure, databases, format and structure handling, descriptor and similarity methods, and classical structure–activity modelling. These themes are still foundational. For example, database and infrastructure work remains a steady backbone of the field. They have, however, been joined, and in volume overtaken, by a new generation of methods.

A defining change is the rise of machine learning and molecular property prediction, which grew from only a handful of articles in 2015 to the single largest topic by 2025. Generative models and de novo molecular design followed a similar trend, emerging around 2019–2020 and expanding rapidly thereafter, while reaction prediction and retrosynthesis consolidated into a distinct theme only in the last few years. Over the same period, some once-prominent topics, such as chemical text mining and named-entity recognition have receded. The figure illustrates this inflection clearly: the centre of gravity of the discipline has moved decisively toward data-driven and generative methods.

## Responding to the rise of AI

This rapid methodological turn is exciting, but it places new demands on how we evaluate the work we publish. The field has reached a point where strong performance on a familiar benchmark is no longer enough on its own. As a community, we can now aim for contributions that generalise: methods accompanied by large-scale, well-curated datasets; rigorous benchmarking against prior studies and careful attribution to the work that came before; as well as honest reporting of limitations and failure rates. The community has grown rightly wary of inflated performance claims arising from data leakage or inappropriate train–test splits, and we will continue to strengthen our guidance accordingly.

These expectations build directly on commitments the current editorial team has already made. Our reproducibility and reusability pilot invited authors with an associated code repository to undergo an enhanced review that checks whether the code is licensed, documented, externally archived, and straightforwardly installable [2]. Our Scientific Contribution Statement asks authors to state, in no more than three sentences, what advances the field and how their work differs from what preceded it; as we noted there, we do not judge articles on novelty alone, but weigh utility, availability, and the nature of the contribution itself [3]. As AI-generated text and code become routine in computational workflows, these safeguards take on renewed importance: ensuring that a reported method is genuine, verifiable, and reusable is now part of what it means to review cheminformatics contributions well.

## Our editorial focus for the coming years

Building on this foundation, and on the trajectory the past decade reveals, we will concentrate our editorial efforts on three priorities:

1. Sustaining Open Science as the default. We will continue to require open data and code, and to develop our reproducibility and reusability review so that published work can be built upon, not merely cited.
2. Setting standards for AI-driven cheminformatics. We will prioritise rigorous benchmarking, large and well-characterised datasets, and transparent evaluation, and we will adapt our review criteria to the realities of generative and AI-assisted research, including questions of data provenance and integrity.
3. Broadening the scope and the community. We will actively solicit work that applies cheminformatics to new molecular modalities and to adjacent disciplines, e.g., via dedicated themed collections, and we will continue to broaden the diversity of our authorship and editorial board so that the Journal reflects the global field it serves.

Cheminformatics has come of age as a driver of molecular discovery and design. Our ambition for *J. Cheminform.* is to remain the venue where the discipline sets its standards and charts its direction. We would also like to express our gratitude to our Managing Editor, Associate Editors, the Editorial Board, reviewers, and authors, and we look forward to the science ahead.

## Data Availability

The bibliographic corpus underlying Fig. 1, the analysis script that produces the figure, and an extended description of the methods are available at https://github.com/BZdrazil/jcheminf-editorial-2026 and archived at Zenodo under https://doi.org/10.5281/zenodo.21474527. The corpus was retrieved from PubMed on 9 June 2026 using the query "Journal of Cheminformatics"[Journal] AND 2015:2025.
